# Sweat sodium composition and sweat loss estimation through wearable sensors and predictive equations in dry and humid hot conditions

**DOI:** 10.3389/fphys.2025.1717275

**Published:** 2026-01-05

**Authors:** David Bandiera, Jean de Bardonnèche, Delphine Margout-Jantac, Léa Dubois, Nisrine El Allaoui, Jonathan Steven Elie Rubio, Jean-Christophe Aubin, Sébastien Racinais, Antonio Tessitore, Yannis Pitsiladis

**Affiliations:** 1 Department of Movement, Human and Health Sciences, University of Rome Foro Italico, Roma, Italy; 2 Environmental Stress Unit, CREPS Montpellier-Font Romeu, Montpellier, France; 3 Qualisud, Université de Montpellier, Avignon Université, CIRAD, Institut Agro, IRD, Université de La Réunion, Montpellier, France; 4 DMEM, Univ Montpellier, INRAE, Montpellier, France; 5 FFTRI, Fédération Française de Triathlon, Saint-Denis, France; 6 Centre for Exercise Science and Medicine (CESAME), Hong Kong Baptist University, Hong Kong SAR, China; 7 Department of Biology, Hong Kong Baptist University, Hong Kong SAR, China

**Keywords:** electrolytes, flame photometer (FP), fluid loss, hydration, recovery, sweating, thermoregulation, whole body sweat rate

## Abstract

**Introduction:**

Individualized sweat testing is essential for tailoring hydration and nutrition strategies, as water and sodium losses during exercise vary greatly across athletes. The validity of a wearable sweat sensor (S1, Flowbio) and a handheld analyzer (LAQUAtwin, Horiba Advanced Techno) for measuring sweat sodium concentration ([Na^+^]) was tested against flame photometry (FP). Additionally, whole body sweat loss (WBSL) estimated by the S1 and by a sweat rate calculator (SRC) was compared to the scale-based method.

**Methods:**

Twenty-three recreationally active participants (11 males, 12 females) completed two sessions in hot and dry (40 °C, 36% rh) and hot and humid (30 °C, 81% rh) controlled environmental conditions on a cycling ergometer (74 ± 12 min, 1.9 ± 0.4 W/kg). Participants were instrumented with two S1 sensors and absorbent patches placed on each upper arm. Sweat was extracted from patches to measure [Na^+^] with LAQUAtwin and FP. Nude body mass was measured to the nearest 0.005 kg before and after exercise, with fluid intake monitored to determine WBSL. The influence of the method and the condition on the measure of sweat [Na^+^] and WBSL was investigated with linear mixed-effects models.

**Results:**

The estimated marginal means of sweat [Na^+^] in dry conditions for S1 and LAQUAtwin were equivalent (both 53 mmol/L, p = 0.952) and significantly lower than FP (63 mmol, both −10 mmol, p < 0.001). No significant interaction effects were observed between methods and conditions. For WBSL, the S1 estimation (1.479 kg) was not different than the scale measure (1.432 kg, 0.047, p = 0.624) while the SRC estimation (1.202 kg) was significantly lower than the scale and S1 (both p < 0.001), without interactions effects.

**Conclusion:**

S1 offers equivalent and more practical collection of sweat [Na^+^] compared to the LAQUAtwin during indoor cycling ergometer exercise. However, measurements from both devices should currently be interpreted with caution and not considered equivalent to laboratory-grade analyses. Furthermore, S1 is an adequate tool during indoor cycling ergometer exercise to estimate WBSL when scale measurements are impractical, while SRC was found to underestimate fluid loss.

## Introduction

During exercise, particularly in hot conditions, sweat production occurs as part of the body’s thermoregulatory response to counteract rising core temperature (Périard et al., 2021). Sweating results in the loss of both water and electrolytes, with sodium being of particular importance due to its role as the primary extracellular osmolyte (Baker and Wolfe, 2020; Shirreffs and Sawka, 2011). To maintain homeostasis, it is recommended to replenish water and sodium during and after exercise (Pohl et al., 2013; Sawka et al., 2007). As water and sweat sodium losses exhibit significant variability among athletes and exercise intensity (Baker et al., 2016), individualized sweat testing is valuable in formulating tailored hydration and nutrition plans (Sawka et al., 2007; Belval et al., 2019; McDermott et al., 2017).

The gold standard technique for quantifying whole-body electrolyte loss is the whole-body washdown method (Shirreffs and Maughan, 1997). However, it requires a cumbersome protocol in a controlled laboratory environment, with restricted exercise modalities (Baker, 2017). Thus, regional sweat collection using patches presents a more practical approach for field studies to gather sweat samples during exercise (Baker, 2017). However, the patch method for measuring sweat sodium concentration ([Na^+^]) has limitations, primarily due to the volume of sweat that needs to be collected. Indeed, sufficient quantities are required for analyses and, depending on the body region, environmental conditions and individual sweat rate, a minimum of ∼15 min after the onset of sweating may be required to collect an adequate sample (Baker et al., 2018). Conversely, leaving the patch on for too long can lead to hidromeiosis or patch saturation, making the measurement invalid (Baker, 2017; Kelly et al., 2016). To ensure reliable results, the patch must be removed from the skin at the optimal time, when it contains an adequate amount of sweat but is not saturated (aiming for ∼0.5–0.7 g for a 5 × 7 cm Tegaderm patch) (Baker et al., 2020). Managing absorbent patch collection on the field can be challenging and requires training to perform high-quality analyses, making implementation difficult for coaches or athletes.

In addition, estimating sweat [Na^+^] typically requires a laboratory analyzer after sweat collection, which provides an average concentration over a set period but does not capture time-related variation. This process requires time and expertise, as it involves transporting sweat samples to the lab, storing samples, performing dilutions, and calibrating the analyzer.

Alternatively, a portable sodium analyzer can be used on-site to measure [Na^+^] directly from the sweat collected, making the process more convenient with a measurement bias of −2 ± 6% (Brown et al., 2024; Goulet et al., 2017). Another option is the Gx Sweat Patch, a thin-film microfluidic patch applied to the forearm that channels sweat into enclosed microtubes, with results accessible via a smartphone app (Baker and Wolfe, 2022). However, its sodium estimates are derived from sweat chloride (mean absolute percent error–MAPE, 5%), which itself is predicted with a MAPE of 15%, ultimately limiting accuracy. Moreover, these approaches require consumable materials and do not provide real-time data. Therefore, there is a need for a valid, reusable, and user-friendly sweat sensor capable of continuously measuring sweat [Na^+^]. Such a device would not only facilitate more responsive hydration strategies during prolonged exercise but also enable the investigation of sweat [Na^+^] kinetics throughout exercise, an aspect that current methods cannot capture.

Also, measuring whole body sweat loss (WBSL) is essential to accurately assess sweat sodium and body water loss. It is well-known that maintaining proper hydration during exercise is essential for optimal performance (Sawka et al., 2007; Racinais et al., 2023). Dehydration can elevate core temperature due to reduced sweating and vasoconstriction of skin vessels, increasing the risk of both performance decline and heat-related illnesses (Périard et al., 2021; Bandiera et al., 2024; Casa et al., 2015). Conversely, overhydration without adequate electrolyte intake can lead to hyponatremia (Périard et al., 2021; Hoffman and Stuempfle, 2015; Montain et al., 2006). WBSL measurement is generally based on nude body mass measurements taken both before and after exercise using a digital scale with a precision of at least 0.1 kg (Cheuvront and Kenefick, 2017), along with tracking fluid intake, fluid loss, urine output, food intake, and nonsweat losses. However, this protocol can be cumbersome and impractical in certain scenarios. Consequently, online sweat rate calculator tools have emerged that estimate fluid loss based on standard variables (e.g., power output, body size, mass, environmental conditions) (Jay et al., 2024a; Jay et al., 2024b; Saltivate, 2024). However, the validity of these tools needs to be established to ensure accurate hydration planning.

Hence, the aim of this study was to test the validity of a continuous wearable sweat sensor for regional sweat [Na^+^] and whole body sweat loss estimation against reference methods in dry and humid hot conditions. The accuracy of the wearable sensor and a field standard handheld analyzer for sweat [Na^+^] was compared to the laboratory gold standard flame photometry analysis (Baker, 2017). Estimations of WBSL from the wearable sensor and from the proposed sweat rate calculator was compared to the gold standard scale method with a 0.005 kg resolution (Cheuvront and Kenefick, 2017). As per previous research, we hypothesized that the LAQUAtwin and the sweat rate calculator would lead similar results to FP and the scale-based method, respectively, whereas S1 study was exploratory.

## Materials and methods

### Participants

Twenty-three participants (11 males and 12 females) volunteered in this study. Participants were recreationally active adults with a mean age of 26.8 ± 7.2 years, a size of 173 ± 9 cm, and a mass of 66.3 ± 9.4 kg. The experimental protocol took place in May and June (i.e., northern hemisphere, end of spring/early summer in Montpellier, France), therefore participants were assumed to be partially heat acclimatized. Of note, the menstrual cycle of the female participants was not controlled, as it was not expected to affect the comparison between devices. All participants gave written informed consent before taking part in the study, which received approval from the University of Montpellier Research Ethics Committee (2023-023) and adhered to the principles of the Declaration of Helsinki.

A power analysis was conducted for the primary comparison (difference in sweat sodium concentration between FP and the S1 device). Assuming a two-sided α = 0.05, 80% power, a minimum detectable mean difference of 150 mg/L (6.5 mmol/L) and an expected standard deviation of the differences of 250 mg/L (11 mmol/L), the required sample size was 22 participants. The sample of 23 participants thus provided adequate statistical power.

### Experimental design

**FIGURE 1:**
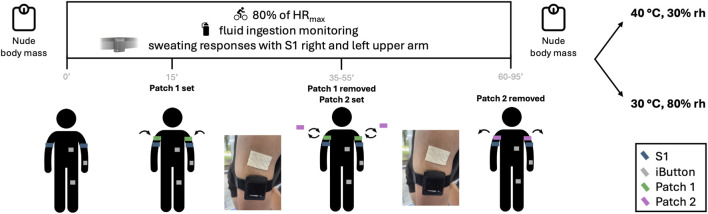
Experimental design of the study. Participants cycled 2 sessions on an ergometer in an environmental chamber in dry and humid hot conditions. Sweating responses were measured continuously with an S1 sensor placed on each upper arm, and absorbent patches were used to collect sweat at 2 time-intervals during exercise to determine sweat odium concentration. Nude body mass was measured before and after exercise, with fluid intake monitored to determine sweat loss.

In a randomized order, participants completed two experimental trials in an environmental chamber at the same time of the day with minimum 24 h of rest in between: one in hot and dry conditions (39.9 °C ± 0.3 °C, 36% ± 3% relative humidity, rh) and one in hot and humid conditions (30.1 °C ± 0.2 °C, 81% ± 1% rh), both with a simulated front wind at 2 m/s. Participants cycled on an ergometer equipped with a power output sensor (Wattbike Pro, Nottingham, United Kingdom) at an intensity corresponding to 80% of their age-predicted maximal heart rate (220 - age), monitored using a H10 chest strap (Polar, Kempele, Finland). The aim was to induce enough sweat over the session to enable the collection of two consecutive patches from each upper arm, with a maximum duration of 95 min (i.e., inducing variations in intensity and time depending on environmental conditions). Also, to ensure a safe exercise progression in hot conditions, skin temperature was measured via iButton hygrochron sensors (Analog Devices, Wilmington, United States) placed on the left lower arm, chest (under the left part of the chest band) and left anterior thigh (Kavitkar and Mehta, 2022), and intestinal temperature was measured via an ingestible pill (e-Celcius, BodyCap, Caen, France) taken 6 h prior the trial (Mougin et al., 2025). A summary of the experimental protocol is found on Figure 1.

### Regional sweat collection

Sweat collection was conducted using the absorbent patch method with 5 × 7 cm Tegaderm + Pad (3M, St. Paul, United States) (Baker, 2017). The absorbent patches were applied to the lateral side of both upper arms after 15 min of exercise. This timing was selected to prevent the collection of initial sweat produced during the gradual transition to steady-state sweating (Baker and Wolfe, 2020; Baker et al., 2020), while also ensuring minimal time for the collection of a sufficient sample volume and comparison with the S1 continuous measure. Prior to patch application, the skin was cleaned with deionized water and dried using sterile gauze (Hartmann, Heidenheim an der Brenz, Germany). Patches were removed upon moderate sweat absorption (aiming for ∼0.5–0.7 g) but before saturation as determined by visual inspection (Baker et al., 2020), with the aim to collect 2 consecutive patches on each arm for each trial. Upon removal of the patch, the absorbent pad was immediately separated from the adhesive dressing using clean forceps and placed in a sterile 10 mL syringe (BD, Franklin Lakes, United States) to extract the sweat from the pad in an air-tight plastic tube (Eppendorf, Hamburg, Germany). The syringe containing the squeezed patch, the plastic tube with the extracted sweat, and the adhesive dressing were weighed on a precision balance to the nearest 0.0001 g (Ohaus corporation, Parsippany, United States) calibrated a week before the experiment (Balco, Saint-Mathieu-de-Tréviers, France) to calculate the mass of the patch prior to extraction (i.e., by subtracting the mass of the syringe and plastic tube). The patch was considered saturated, and the sweat sample deemed unreliable, if its mass exceeded 1.4000 g: 0.7 g of sweat +0.7 g for the mass of a clean patch.

### S1 wearable

**FIGURE 2:**
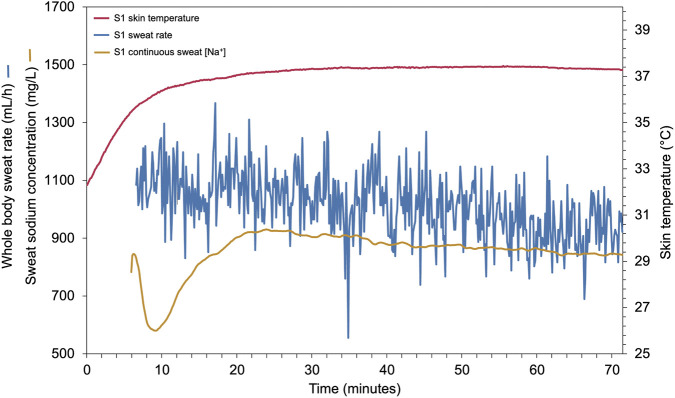
Example of S1 real-time recordings during an indoor cycling session with continuous whole body sweat rate (mL/h, blue), upper arm sweat sodium concentration (mg/L, orange) and skin temperature (°C, red, right axis). The initial peak of sweat sodium concentration is due to the presence of residues on the skin and was excluded before analysis.

The S1 (Flowbio, London, United Kingdom) is a wearable (42.6 × 50.6 × 13.8 mm, 18 g), reusable electrochemical sensor that uses microfluidics to capture sweat from the skin (Supplementary Material 1). The internal volume is inferior to 2 μL, providing capture times (from sweating to sensor reading) of less than 3 min. The S1 employs a conductometric sensor to measure sodium concentration in sweat. A skin temperature sensor is present on the device, as well as an internal humidity and temperature sensor. WBSL is determined by a proprietary algorithm from a combination of physically acquired metrics (heart rate, skin temperature, intensity, accelerometry) and ambient parameters (temperature, humidity). [Na^+^], WBSL and skin temperature are measured and recorded in real-time with a time interval set to 4 s (Figure 2).

S1 devices were fastened with a strap on both cleaned upper arms below the deltoid tuberosity on a flat surface allowing full contact between the sensor and the skin. The sensors were started few minutes before pedaling and were stopped at the end of the exercise. For comparing [Na^+^] of the S1 (continuous reading) with the FP and LAQUAtwin (2 reading per upper arm corresponding to the 2 patches), the average [Na^+^] measured by the S1 was calculated over the time-interval during which the patches remained on the skin. Two [Na^+^] segments of the S1 corresponding to the two time-intervals were extracted and averaged, resulting in two sweat [Na^+^] values per S1. Before further analysis, each segment was observed individually to check for operating errors: lack of values for several minutes, sudden non-physiological drop of sweat [Na^+^], important noise. These irregularities were often caused by sensor movement on the skin, allowing the introduction of an air bubble between the skin and the sensor. For WBSL estimation, the S1 was paired with a Garmin Forerunner 255 watch (Garmin, Olathe, United States). WBSL was afterward corrected for nonsweat loss estimated at 0.20 g/kcal (Cheuvront and Kenefick, 2017), with energy expenditure (kcal) estimated from the watch. For subsequent analysis, the WBSL of the entire session was extracted and compared with the reference scale method.

Finally, the comfort of the sensor was assessed at the end of each session. Participants completed a visual analog scale (VAS) ranging from “The sensor did not bother me at all during the exercise” to “The sensor bothered me a lot during the exercise”. The VAS was 10 cm long, with higher values indicating greater discomfort.

### LAQUAtwin Na-11 portable device

The portable sodium analyzer LAQUAtwin-Na-11 (Horiba Advanced Techno, Kyoto, Japan) was employed as a “field standard” to assess sweat [Na^+^]. Sweat samples were analyzed in temperate environments with the LAQUAtwin just after sweat extraction from the patch. Prior to each session, the device was calibrated following the manufacturer’s guidelines. After switching on the device, it was rinsed with deionized water and dried using gauze, and a two-point calibration was conducted using 150 ppm and 2000 ppm standards. Between each sample analysis, the sensor was cleaned with deionized water and dried with a gauze. For each analysis, the plastic tube containing the sweat was shaken to ensure a homogeneous sample. A drop of sweat was then collected with a micropipette (Gilson, Middleton, United States) and placed on the LAQUAtwin sensor. This process was repeated three times (triplicates), and the average [Na^+^] was recorded in mg/L. The calculation of a coefficient of variation for each sample was performed. Because sodium was present in the collection materials (e.g., patch, syringe, plastic tube), background sodium was quantified by running deionized water through the entire protocol five times (Baker et al., 2018). This procedure yielded a residual sodium concentration of 103 ± 7 mg/L (4.5 ± 0.3 mmol/L), which was subsequently subtracted from all LAQUAtwin measurements.

### Flame Photometry

The analytical reference assay method used for sodium quantification was flame atomic emission spectrometry (FP) (Baker, 2017). Errors mainly caused by variations in instrumental parameters and/or matrix effects that may impair accuracy and/or precision were evaluated and considered in the results obtained. The absence of matrix effects was verified, and the external calibration method was adopted. A calibration range was prepared using an appropriate standard solution dilution of 1000 mg/L. The solutions were diluted in a mixture of water for analysis, 1% dilute nitric acid to increase atomization of sodium and cesium to attenuate instrumental interference (buffer ionization) into the flame. The emission intensities from sodium (I^Na^) were measured 3 consecutive times (triplicates) at 586.9 nm, and the average [Na^+^] was recorded in mg/L. The calculation of a coefficient of variation for each sample was performed. External standard calibration curves were used by plotting I^Na^ versus [Na^+^] for analytical solutions in the 0.25–1.5 mg/L range. When the I^Na^ of the sweat samples was outside of the range of calibration, samples were excluded. Also, several three-level quality control (QC) samples were prepared and analyzed using the calibration line to ensure the accuracy of the assay method. If the mean difference between the I^Na^ of the QC samples and their expected value exceeded 10% of the I^Na^ of the sweat samples, the samples were excluded from the analysis. Finally, to exclude sodium present in the collection materials, the background sodium in the FP protocol was calculated to be 94 ± 5 mg/L (4.1 ± 0.2 mmol/L), this value was therefore subtracted from the FP measurement (Baker et al., 2018).

### Scale-based method for WBSL

WBSL was determined by assessing the change in pre-to post-exercise nude body mass, corrected for fluid intake and nonsweat loss, as: mass pre (kg) + fluid ingested (L) - mass post (kg) - nonsweat loss (L), with the reasonable assumption that 1 L of sweat = 1 L of water = 1 kg. Nonsweat sodium loss was estimated at 0.20 g/kcal (Cheuvront and Kenefick, 2017), with energy expenditure derived from the watch, consistent with the S1 method. As a reference method, nude body mass was measured both before and after exercise using a digital scale with a resolution of 0.005 kg and a linearity of ±0.015 g (Kern & Sohn GmbH, Balingen, Germany), and participants towel dried prior to each measurement (Cheuvront and Kenefick, 2017). During the exercise, participants were not allowed to go to the toilet, to evacuate saliva, nor eat any food. To minimize the risk of hypohydration, participants were allowed to consume water *ad libitum* during the experimental sessions. Water bottle mass from pre-to post-exercise was measured with a resolution of 0.001 kg using a compact digital scale (Terraillon, Croissy sur Seine, France) to calculate fluid intake.

### Sweat rate calculator

WBSL was also estimated with an online sweat rate calculator (SRC) developed by Jay et al., in 2024 (Jay et al., 2024b) (https://sweatratecalculator.com/hydration-calculator). This calculator allows to estimate the sweat rate for an indoor cycling exercise from a combination of metrics: body height, body mass, air temperature, air humidity, wind speed, power output and duration of the exercise. It is a tool easily accessible online, with a validity of 0.01 L/h, with 95% confidence interval comprised between −0.22 L/h and 0.25 L/h (Jay et al., 2024b).

### Statistics

First, the differences in exercise responses between the dry and humid conditions were investigated, including exercise duration, average heart rate, average power output and average core and skin temperature. Depending on data distribution assessed by a Shapiro-Wilk test, a Student’s t-test (t, parametric) or Wilcoxon test (W, non-parametric), was respectively performed to investigate paired differences between conditions. In addition, exploratory Pearson correlation analyses were performed to examine potential associations between the average intestinal temperature, regional sweat [Na^+^] (average of the exercise measured with FP) and WBSL (scale-based method).

The validity of the S1 and the LAQUAtwin were evaluated against the FP, and for WBSL, S1 and SRC were compared to the scale-based method. To detect differences between methods and environmental conditions (dry and humid), a linear mixed-effects model was fitted using method and condition as fixed effects, and participant as a random factor. This structure accounted for repeated measures within participants and unbalanced data. Pairwise comparisons were adjusted using the Tukey method. In addition, Bland-Altman plots were performed for graphical agreement assessment, pooling both environmental conditions together (Bland and Altman, 1986). The mean bias (paired systematic difference) and the 95% limits of agreement (LOA = bias ±1.96 * standard deviation of the differences) were calculated for all comparisons. The concordance correlation coefficient (CCC) was also calculated between compared methods (Lawrence, 1989).

All statistics were conducted in R statistical software (version 4.4.1) (R Core Team, 2024). Values for sweat [Na^+^] were converted from mg/L to mmol/L by dividing by the atomic mass of sodium (22.989769 g/mol). Data were presented in mean ± standard deviation apart for the results of the linear mixed-effects model reported in estimated marginal means ± standard error. The level of significance was set at p < 0.05.

## Results

### Exercise responses

Exercise responses in dry and humid hot conditions are presented in Table 1. In dry condition, exercise duration (75 ± 13 min), average heart rate (157 ± 9 bpm), average power output (126 ± 30 W), and average core temperature (38.4 °C ± 0.3 °C) were similar to humid condition (non-significant differences). In contrast, average skin temperature was significantly higher in dry (36.5 °C ± 0.5 °C) compared to humid condition (35.3 °C ± 0.6 °C, p < 0.001).

**TABLE 1:** Exercise responses comparison in dry (40 °C and 36%) and humid (30 °C and 81%) hot conditions. Statistic refers to the test value obtained from either a paired Student’s t-test (t: parametric) or a Wilcoxon signed-rank test (W: non-parametric), depending on data distribution. Values are expressed in mean ± standard deviation.

Parameter	Dry	Humid	Difference	Statistic	P-value
Duration (min)	75 ± 13	72 ± 11	3 ± 10	t = 0.7	0.467
Heart rate (bpm)	157 ± 9	158 ± 9	−1 ± 6	t = −0.4	0.662
Power output (W)	126 ± 30	133 ± 32	−7 ± 10	t = −0.7	0.510
Average core temp (°C)	38.4 ± 0.3	38.3 ± 0.4	0.1 ± 0.4	W = 117	0.972
Average skin temp (°C)	36.5 ± 0.5	35.3 ± 0.6	1.2 ± 0.7	t = 7.2	<0.001

Exploratory correlation analyses revealed no significant associations between markers of heat strain and indices of fluid or sodium loss. Specifically, regional sweat [Na^+^] (r = 0.09, p = 0.58) and WBSL (r = −0.23, p = 0.13) were not correlated with average intestinal temperature. Similarly, regional sweat [Na^+^] and WBSL were not significantly correlated (r = 0.11, p = 0.51).

### Sample exclusion and sensor operation

**FIGURE 3:**
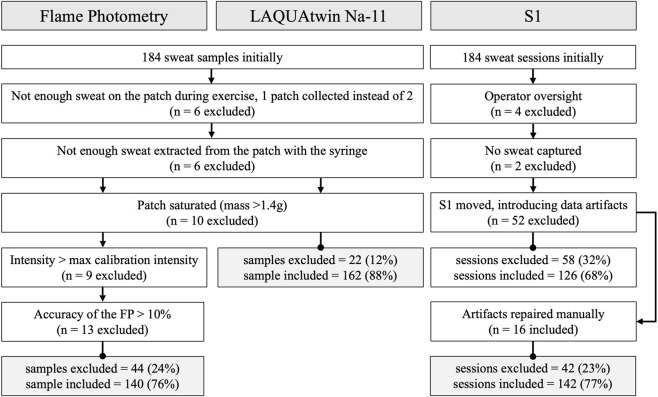
Sample exclusion criteria for the Flame Photometry and LAQUAtwin, and session exclusion criteria for the S1 device. Twenty-three participants completed two cycling sessions, during which sweat composition was analyzed twice on each upper arm (23*2*2*2 = 184), for a target of 184 samples (for LAQUAtwin and FP) and 184 sessions (for S1).

In total, 23 participants completed two cycling sessions, during which sweat composition was analyzed twice on each upper arm (23*2*2*2 = 184), for a target of 184 samples (for LAQUAtwin and FP) and 184 sessions (for S1). Forty-four samples were excluded from FP analysis and 22 for LAQUAtwin, due to not enough sweat being extracted from the patch, the patch being saturated, or the measure being considered not valid (details in Figure 3). For S1, 16 sessions were manually repaired after the removal of artefacts, and a total of 42/184 sessions were excluded. These S1 excluded sessions were mainly caused by sensor movement. Out of the 46 exercise sessions performed by participants, 23 included complete sweat [Na^+^] data from all three devices (i.e., FP, LAQUAtwin and S1). For WBSL, all 46 sessions provided valid data for the scale-based method, the S1 and the SRC, and none were excluded. Wearing the S1 did not seem to be associated with discomfort, as VAS scores indicated that participants were not distracted or bothered by the sensor: 0.4 ± 0.6 cm out of 10 cm. Also, the sensor took an average of 8.3 ± 3.5 min after the beginning of the exercise to display the first sweat [Na^+^] value.

### Sweat [Na^+^] measures

The coefficient of variation of the triplicates for FP was 0.8% ± 0.7%, and the mean difference between the QC samples I^Na^ and their expected value represented 2.1% ± 4.0% of the sweat samples I^Na^. For LAQUAtwin, the coefficient of variation of the triplicates was 0.6% ± 1.3%.

The linear mixed-effects model with participant as a random intercept revealed significant main effects of device and environmental condition on sweat [Na^+^] (p < 0.001). The estimated marginal mean of sweat [Na^+^] in dry conditions was 63 mmol/L for the FP device. S1 and LAQUAtwin significantly underestimated sweat [Na^+^] compared with FP (both 53 mmol/L), each by 10 mmol/L (p < 0.001, Table 2). As expected, the difference between S1 and LAQUAtwin was not significant (0 mmol/L, p = 0.952).

**TABLE 2:** Sweat sodium concentration comparison between flame photometry (FP), the S1 sensor, and LAQUAtwin in dry and humid hot conditions. A linear mixed-effects models was fitted using method and condition as fixed effects, and participant as a random factor. Results are presented in estimated marginal means ± standard error.

Comparison	Condition	Method	n	Estimated sweat [Na^+^]	Estimated error	P-value
				(mmol/L)	(mmol/L)	
FP - S1	*dry*	FP	71	63 ± 4	10 ± 2	<0.001
		S1	70	53 ± 4		
	*humid*	FP	69	60 ± 4	9 ± 2	<0.001
		S1	72	51 ± 4		
FP - LAQUAtwin	*dry*	FP	71	63 ± 4	10 ± 1	<0.001
		LAQUAtwin	80	53 ± 4		
	*humid*	FP	69	60 ± 4	8 ± 1	<0.001
		LAQUAtwin	82	52 ± 4		
LAQUAtwin - S1	*dry*	LAQUAtwin	80	53 ± 4	0 ± 2	0.952
		S1	70	53 ± 4		
	*humid*	LAQUAtwin	82	52 ± 4	−1 ± 1	0.737
		S1	72	51 ± 4		

Sweat [Na^+^] measured by FP was slightly lower in the humid condition (60 mmol/L, p = 0.034) compared to the dry one, and no significant interactions between device and condition were detected (p = 0.44 for S1*humid and p = 0.27 for LAQUAtwin*humid).

**FIGURE 4:**
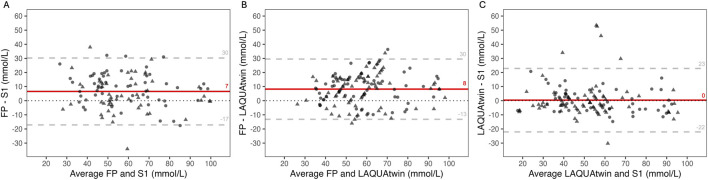
Bland-Altman graph of sweat sodium concentration measured in dry (circle) and humid (triangle) hot conditions by flame photometry (FP) and the S1 sensor (panel **(A)**), FP and LAQUAtwin (panel **(B)**), and LAQUAtwin and S1 (panel **(C)**). Red line: mean error or “bias”, dashed grey lines: 95% limits of agreement, dotted black line: zero difference.

In addition, when both dry and humid conditions were pooled and only paired data were compared, the bias was 7 ± 12 mmol/L for S1 (55 ± 19 mmol/L) compared to FP (62 ± 18 mmol/L), with LOA comprised between −17 and 30 mmol/L (Figure 4A) and a CCC of 0.74. For LAQUAtwin (54 ± 16 mmol/L) compared to FP (62 ± 17 mmol/L), the bias was 8 ± 11 mmol/L with LOA comprised between −13 and 30 mmol/L (Figure 4B) and a CCC of 0.69. For S1 (53 ± 21 mmol/L) compared to LAQUAtwin (54 ± 20 mmol/L) the bias was 0 ± 11 mmol/L with LOA comprised between −22 and 23 mmol/L (Figure 4C) and a CCC of 0.84.

### Side-to-side variability

FP sweat [Na^+^] measured on the right (61 ± 16 mmol/L) and left (64 ± 18 mmol/L) upper arms showed a bias of −3 ± 8 mmol/L. S1 right (55 ± 21 mmol/L) and left (51 ± 21 mmol/L) measures presented a bias of 4 ± 8 mmol/L while for LAQUAtwin the right (55 ± 18 mmol/L) and left (55 ± 19 mmol/L) side had a bias of 0 ± 4 mmol/L. Also, the temperature measured with the S1 on the right (37.3 °C ± 1.5 °C) and on the left (37.3 °C ± 1.5 °C) upper arms revealed a bias of 0.0 °C ± 0.3 °C.

### Whole body sweat loss measures

In average, participants drank 1.002 ± 0.403 L of water, and nonsweat loss represented 0.132 ± 0.023 L. Participants lost 0.64% ± 0.57% of their body mass during exercise (overconsumption of water and mass gain in 5/46 trials), with individual changes ranging from a 1.88% loss to a 0.68% gain.

The linear mixed-effects model revealed significant main effects of device and environmental condition on WBSL (p < 0.001). The estimated marginal mean of WBSL in dry conditions was 1.432 kg for the scale-based method. The estimation for the S1 (1.479 kg) was not different (−0.047, p = 0.624) while the estimation for the SRC (1.202 kg) was significantly lower than the scale and S1 (0.230 and 0.277 kg respectively, both p < 0.001, Table 3).

**TABLE 3:** Whole body sweat loss (WBSL) comparison between the scale-based method (Scale), the S1 sensor, and the sweat rate calculator (SRC) in dry and humid hot conditions. A linear mixed-effects models was fitted using method and condition as fixed effects, and participant as a random factor. Results are presented in estimated marginal means ± standard error.

Comparison	Condition	Method	Estimated WBSL	Estimated error	P-value
			(kg)	(kg)	
Scale - S1	*dry*	Scale	1.432 ± 0.055	−0.047 ± 0.051	0.624
		S1	1.479 ± 0.055		
	*humid*	Scale	1.202 ± 0.055	0.081 ± 0.051	0.251
		S1	1.121 ± 0.055		
Scale - SRC	*dry*	Scale	1.432 ± 0.055	0.230 ± 0.051	<0.001
		SRC	1.202 ± 0.055		
	*humid*	Scale	1.202 ± 0.055	0.362 ± 0.051	<0.001
		SRC	0.840 ± 0.055		
S1 - SRC	*dry*	S1	1.479 ± 0.055	0.277 ± 0.051	<0.001
		SRC	1.202 ± 0.055		
	*humid*	S1	1.121 ± 0.055	0.281 ± 0.051	<0.001
		SRC	0.840 ± 0.055		

Across all devices, WBSL was lower in the humid condition by 0.229 kg (p < 0.001). No significant interactions between device and condition were detected (p = 0.077 for S1*humid and p = 0.068 for SRC*humid), indicating that S1 and SRC deviation to the scale was consistent across environmental conditions.

**FIGURE 5:**
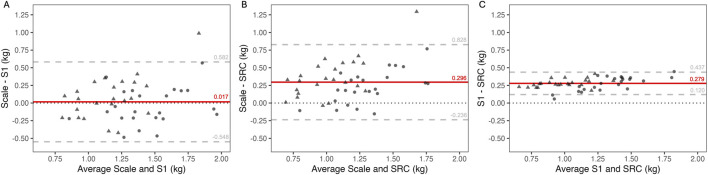
Bland-Altman graph of whole body sweat loss measured in dry (circle) and humid (triangle) hot conditions with the scale-based method (scale) and the S1 sensor (panel **(A)**), the scale and the sweat rate calculator (SRC) (panel **(B)**), and the S1 and SRC (panel **(C)**). Red line: mean error or “bias”, dashed grey lines: 95% limits of agreement, dotted black line: zero difference.

In addition, when both environmental conditions were pooled and only paired data were compared, the bias for WBSL was 0.017 ± 0.288 kg (0.038 ± 0.260 L/h, results expressed in sweat rate in Supplementary Material 2) for S1 (1.300 ± 0.299 kg) compared to the scale (1.317 ± 0.357 kg) with LOA comprised between −0.548 and 0.582 kg (Figure 5A), and a CCC of 0.62. For SRC (1.021 ± 0.259 kg) compared to the scale (1.317 ± 0.357 kg), the bias was 0.296 ± 0.271 kg (0.268 ± 0.272 L/h, SM2) with LOA comprised between −0.236 and 0.828 kg (Figure 5B), and a CCC of 0.43. For SRC (1.021 ± 0.259 kg) compared to S1 (1.300 ± 0.299 kg), the bias was 0.279 ± 0.081 kg (0.229 ± 0.064 L/h, SM3) with LOA comprised between 0.120 and 0.437 kg (Figure 5C), and a CCC of 0.64.

## Discussion

The validity of the S1 sensor and portable analyzer LAQUAtwin to measure sweat [Na^+^] was tested against the gold standard FP. Although the S1 and LAQUAtwin devices produced equivalent results, both significantly underestimated sweat [Na^+^] relative to FP. Therefore, while the S1 may offer practical advantages for field data collection compared to the LAQUAtwin, its measurements should currently be interpreted with caution and not considered equivalent to laboratory-grade analyses. However, for WBSL estimation, the errors of the S1 compared to the gold standard scale method were not significant and considered acceptable for fluid intake recommendation in indoor cycling, whereas WBSL obtained from the sweat rate calculator was significantly different from the gold standard and should be used with caution in the environmental conditions tested.

### Exercise responses

The exercise duration and intensity were similar in dry (40 °C and 36%) and humid (30 °C and 81%) conditions (Table 1), suggesting comparable metabolic heat production (Périard et al., 2021). However, despite similar core temperature, skin temperature was lower in the humid compared to the dry condition, indicating a lower heat stress in the humid condition (Table 1) (Cramer and Jay, 2019). Indeed, the 2 m/s airflow likely reduced heat strain in the humid environment by enhancing sweat evaporation and heat dissipation, whereas in the dry condition it may have increased convective heat gain because air temperature (40 °C) exceeded skin temperature (36.5 °C) (Foster et al., 2022). This reduced heat stress in the humid condition likely resulted in a lower WBSL.

Furthermore, the significant lower sweat [Na^+^] observed in the humid condition likely reflects the reduced WBSL, as these two parameters are closely related (Buono et al., 2008). Indeed, previous work has shown that higher sweat rates are generally associated with higher sweat [Na^+^] within individuals. This relationship is thought to result from reduced sodium reabsorption by eccrine sweat glands when secretion rate increases. However, this phenomenon is mainly observable at the intra-individual level. This may explain why, in the present study, when data from both conditions were pooled, the correlation between sweat [Na^+^] and WBSL was not significant. In addition, the reduced WBSL could also be related to effects of hidromeiosis in the humid condition. Indeed, high skin wittedness due to a reduced sweat evaporation can lead to lower sweat eccrine secretion and lower sweat [Na^+^] (Baker, 2017).

### Sweat [Na^+^] measures

The linear mixed-effect model analysis revealed no significant interaction between the method used to measure sweat [Na^+^] (FP, S1, LAQUAtwin) and the environmental conditions (dry and humid hot conditions). These results indicate that S1 and LAQUAtwin errors relative to FP were consistent across both climates. When both conditions were considered, the differences between S1 and LAQUAtwin over the gold standard FP revealed biases of 10% ± 22% and 12% ± 17%, respectively (Figures 4A,B). The magnitude and the variability of these differences do not support the use of these methods for precise measures of sweat [Na^+^] (Barnes et al., 2019). However, there was no significant difference between S1 and LAQUAtwin (0 and −1 mmol/L in dry and humid conditions respectively, Table 2). Therefore, S1 provides in average an equivalent ability to LAQUAtwin for determining sweat [Na^+^] in athletes during indoor cycling exercise.

To assess the potential impact of S1 and LAQUAtwin errors on sodium replacement strategies, regional sweat [Na^+^] values obtained from each device were converted into whole-body sodium losses using a scaling (Baker et al., 2020), together with the fluid losses of the present study measured via the scale-based method (details of the calculations in Supplementary Material 3). This approach allowed to estimate how inaccuracies from S1 and LAQUAtwin in sweat [Na^+^] measurement may translate into practical recommendations deviations for sodium intake after exercise. In our study, 95% of the S1 errors relative to FP fell between an overestimation of 685 mg and an underestimation of 909 mg of whole-body sodium loss. For the LAQUAtwin, errors ranged from an overestimation of 616 mg to an underestimation of 897 mg. To our knowledge, no clear consensus exists regarding the exact amount of sodium that should be replaced following exercise (Barnes et al., 2019), making it difficult to define a universally acceptable error margin. Nevertheless, assuming that total sodium losses should be replaced, these error ranges correspond to less than half a teaspoon of table salt (one teaspoon contains approximately 2300 mg of sodium). From a practical standpoint, for the type of exercise duration (75 min), intensity (1.9 W/kg), and environmental conditions tested in this study, such deviations are unlikely to lead to meaningfully incorrect sodium replacement strategies. Consequently, both the S1 and LAQUAtwin appear acceptable for estimation of sodium intake after exercise.

Beyond the accuracy of the measure itself, the practical aspects associated with the use of FP, LAQUAtwin, and S1 should be considered by practitioners. The FP method involves a labor-intensive analytical process, including optimal sweat collection in patches, extraction, sample transport to the laboratory, storage, dilution, instrument calibration, and manual analysis (Baker, 2017). The LAQUAtwin, although portable, still requires patch handling, a precision balance, syringe use, and pipetting. In contrast, the S1 offers a simple “plug-and-play” approach, providing immediate readings of sweat [Na^+^] without additional sample processing. As the S1 and LAQUAtwin demonstrated equivalent performance (Figure 4C) and was not impacted by hot and humid conditions, the S1 appears particularly suitable for field use due to its practicality, whereas the FP should be prioritized when the highest analytical accuracy of sweat [Na^+^] is required.

The measurement reliability of the S1 and LAQUAtwin was estimated by the comparison of sweat [Na^+^] between the left and right upper arms. Indeed, side-to-side variation in sweat [Na^+^] is typically low (0–3 mmol/L) (Baker et al., 2018). For S1 and LAQUAtwin, biases of 5% ± 28% and 0% ± 8% were respectively found. Hence, side-to-side reliability of both methods are considered adequate for tracking intra-individual adaptations during heat acclimation, as sweat [Na^+^] typically decreases by 30%–50% in acclimated individuals (Allan and Wilson, 1971; Chinevere et al., 2008; Tyler et al., 2016). Also, given that the day-to-day variability in sweat [Na^+^] is approximately 15%, the S1 and LAQUAtwin are considered suitable for monitoring substantial fluctuations due to factors such as exercise intensity, environmental conditions, and clothing (Baker, 2017).

A previous version of the LAQUAtwin (B-722) demonstrated a bias of 1 mmol/L and a standard error of the estimate of 3.8 mmol/L when validated against ion chromatography (IChr) (Goulet et al., 2017), hence our former hypothesis on the validity of the LAQUAtwin. However, it was not supported in our study, where a bias of 8 mmol/L was observed. One possible explanation is the different reference method used (i.e., IChr compared to FP), which can affect sweat [Na^+^] measurements (Goulet et al., 2017; Dziedzic et al., 2014). In comparison, the LAQUAtwin utilizes a direct ion-selective electrode (DISE), which tends to be 11% lower than those obtained from frozen sweat samples analyzed by FP (Dziedzic et al., 2014), which aligns well with the 12% bias observed in our study.

The limitation of our sweat [Na^+^] analysis is the absence of crossover measurements to directly assess the magnitude of differences between the analytical methods used (Dziedzic et al., 2014) (i.e., direct ion-selective electrode for LAQUAtwin, atomic emission spectroscopy for FP, and electrochemical sensing for S1). Furthermore, each device presents its own inherent bias, and the conditions under which the measurements were performed likely influenced the sweat [Na^+^] values. Indeed, the analyses were conducted inside the chamber for S1, immediately after patch removal in a temperate environment for LAQUAtwin, and several days later following sample freezing for FP (Baker, 2017). These limitations are inherent to the protocols used and may partly explain the differences observed between methods.

### Whole body sweat loss measures

S1 estimation of WBSL in hot and dry conditions was not significantly different than the sweat loss measured with the scale-based method (−0.047 kg, p = 0.624, Table 3), and no significant interaction was observed with humid conditions. When dry and humid conditions were considered together, 95% of the differences ranged between an underestimation of 0.582 kg and an overestimation of 0.548 kg, corresponding to −0.9%–0.8% of body mass difference in our participants (Figure 5A). Hence, excluding nonsweat losses, the maximum errors of the sensor remained within the recommended 2% threshold to avoid deleterious effects of dehydration (Cheuvront et al., 2003). Therefore, the S1 is considered as a valuable tool for estimating WBSL on an indoor cycling ergometer. It offers an effective solution for optimizing hydration during training when precise scale measurements are not applicable.

In contrast, the SRC in dry and humid hot conditions estimated a significantly lower WBSL than the scaled-based method and the S1 (−0.230 and −0.277 kg respectively, both p < 0.001, Table 3), conversely to our hypothesis. This finding contrasts with the original validity study, where the bias from the scale-based method during an indoor cycling exercise was 0.01 L/h (Jay et al., 2024b) (0.268 ± 0.272 L/h, SM3). This discrepancy could be attributed to the particularly strenuous environmental conditions in our study, either very hot (40 °C) or highly humid (80%), while the SRC has been validated in temperatures ranging from 23 °C to 40 °C and humidity levels between 14% and 60%. Our conditions were therefore at the upper limits or outside these ranges, and it may have led to reduced heat dissipation and higher sweat rate (Périard et al., 2021) than what was predicted by the SRC. Of note, an underestimation of sweat loss may be less problematic than overestimation, as the latter could encourage excessive fluid intake and increase the risk of exercise-associated hyponatremia (Montain et al., 2006). Ninety-five percent of the differences ranged between an underestimation of 0.828 kg and an overestimation of 0.236 kg, corresponding to −1.2%–0.4% of body mass difference in our participants. The errors of the SRC therefore remained within the recommended 2% threshold (Cheuvront et al., 2003).

#### Limitations

The present study was conducted in under specific experimental conditions, with a moderate intensity indoor cycling exercise performed in controlled environmental settings and *ad libitum* fluid intake. The accuracy and practical implications reported here should therefore be interpreted within the context of these conditions only. Of note, the hydration status of the participants was not controlled prior to the trials. In addition, this study focused on validating the S1 sensor when placed on the upper arm, therefore, its accuracy at other body sites cannot be inferred. Future studies testing the S1 in outdoor field-conditions are now warranted.

## Conclusion

The S1 wearable is a sweat sensor allowing continuous sweat sodium concentration ([Na^+^]) and whole body sweat loss (WBSL) estimation. Our study demonstrated a significant difference for sweat [Na^+^] measure of the S1 and the LAQUAtwin when compared to the gold standard flame photometry. However, S1 and LAQUAtwin measurements yielded comparable results. S1 appears to offer a more practical alternative to the LAQUAtwin since it does not necessitate absorbent patch application. Additionally, our study showed that the S1 is adequate to estimate WBSL during cycling ergometer exercise when the gold standard scale-based method is not feasible, while the sweat rate calculator measures were significantly lower than the scale and should therefore be used with caution in very dry or humid conditions. In summary, the S1 is a promising tool when tested in laboratory setting, with additional studies warranted to assess its validity and reliability in field conditions.

## Data Availability

The raw data supporting the conclusions of this article will be made available by the authors, without undue reservation.
