# Supplementary material for: Sweat sodium composition and sweat loss estimation through wearable sensors and predictive equations in dry and humid hot conditions
Source: Front Physiol. 2026 Jan 5;16:1717275. doi: 10.3389/fphys.2025.1717275 (PMC12813213; doi:10.3389/fphys.2025.1717275)
Supplement: Supplementary file 1 [file DataSheet1.pdf]

Supplementary materials

"Sweat sodium composition and sweat loss estimation through wearable sensors and predictive equations in dry and humid hot conditions"

SM1: Features of the S1 sensor. The sensor is positioned with its back against the skin, the biochemical sensors are located inside the sweat duct.

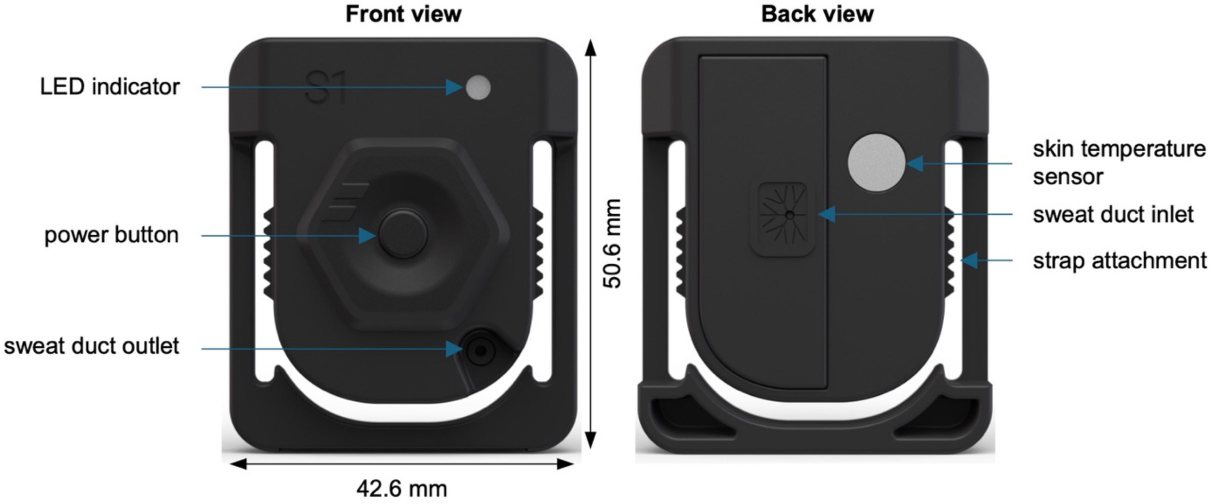

SM2: Bland-Altman graph of sweat rate (L/h) measured in dry (circle) and humid (triangle) hot conditions with the scale-based method (scale) and the S1 sensor (panel A), the scale and the sweat rate calculator (SRC) (panel B), and the S1 and SRC (panel C). The shaded area represents ± 2% of body mass (BM) loss. Red line: mean error or "bias", dashed grey lines: 95% limits of agreement, dotted black line: zero difference.

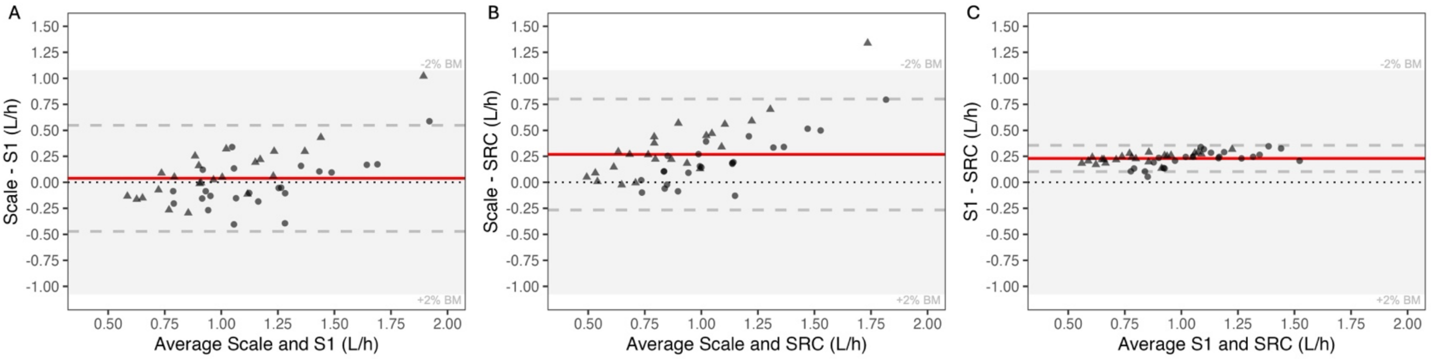

	Mean (L/h)	n	Mean Error	LOA upper	LOA lower
**Scale**	1.112 ± 0.404	46	0.038 ± 0.260	0.548	-0.472
**S1**	1.074 ± 0.256				
**Scale**	1.112 ± 0.404	46	0.268 ± 0.272	0.801	-0.266
**SRC**	0.845 ± 0.225				
**S1**	1.074 ± 0.256	46	0.229 ± 0.064	0.356	0.103
**SRC**	0.845 ± 0.225				

SM3: Potential impact of S1 and LAQUAtwin errors on sodium replacement strategies.

Regional sweat [Na^+] values obtained from S1 and LAQUAtwin devices were converted into whole-body sodium losses (in mg) using a scaling equation and fluid losses of the present study measured via the scale-based method.

1. Regional sweat [Na^+] 95% error (mmol/L):

	LOA -	BIAS	LOA +
**FP-S1**	-17	7	30
**FP-LAQUATWIN**	-13	8	30
**LAQUATWIN-S1**	-22	0	23

2. Whole-body sweat [Na^+] - 95% error (mmol/L):

	LOA -	BIAS	LOA +
**FP-S1**	-23	17	30
**FP-LAQUATWIN**	-20	18	30
**LAQUATWIN-S1**	-25	0	26

Based on the scale up equation from upper arm sweat [Na^+] to whole-body sweat [Na^+] cross validated in Baker et al. 2020:

$$\text{Whole-body sweat } [Na^+] = 0.565*(\text{upper arm sweat } [Na^+])+12.927$$

Baker LB, Nuccio RP, Reimel AJ, Brown SD, Ungaro CT, De Chavez PJD, et al. Cross-validation of equations to predict whole-body sweat sodium concentration from regional measures during exercise. Physiol Rep. 2020;8(15). Available from: https://onlinelibrary.wiley.com/doi/abs/10.14814/phy2.14524

3. Whole-body sweat [Na^+] - 95% error (mg/L):

	LOA -	BIAS	LOA +
**FP-S1**	-520	383	690
**FP-LAQUATWIN**	-468	403	681
**LAQUATWIN-S1**	-584	0	594

[Na^+] in mg/L = ([Na^+] in mmol/L) * 22.989769

4. Whole-body sweat sodium losses - 95% error (mg):

	LOA -	BIAS	LOA +
**FP-S1**	-685	504	909
**FP-LAQUATWIN**	-616	531	897
**LAQUATWIN-S1**	-769	0	783

The value of whole-body sweat [Na^+] (mg/L) was multiplied by the mean WBSL: 1.317 L.
